# A Delta–Omicron Bivalent Subunit Vaccine Elicited Antibody Responses in Mice against Both Ancestral and Variant Strains of SARS-CoV-2

**DOI:** 10.3390/vaccines11101539

**Published:** 2023-09-28

**Authors:** Tiantian Wang, Jing Zheng, Huifang Xu, Zhongyi Wang, Peng Sun, Xuchen Hou, Xin Gong, Bin Zhang, Jun Wu, Bo Liu

**Affiliations:** 1 Department of Microorganism Engineering, Beijing Institute of Biotechnology, Beijing 100071, China; con_an@126.com (T.W.); 18845789169@163.com (H.X.); zhongyi_wang@foxmail.com (Z.W.); sunpeng990718@163.com (P.S.); hoxuch@163.com (X.H.); 13693331905@163.com (X.G.); zb008513@126.com (B.Z.); 2 Xiamen Center for Disease Control and Prevention, Xiamen 361000, China; zhengjing1103@foxmail.com

**Keywords:** SARS-CoV-2, RBD, subunit vaccine, bivalent vaccine, glycoengineered yeast

## Abstract

Continued mutation of the SARS-CoV-2 genome has led to multiple waves of COVID-19 infections, and new variants have continued to emerge and dominate. The emergence of Omicron and its subvariants has substantially increased the infectivity of SARS-CoV-2. RBD genes of the wild-type SARS-CoV-2 strain and the Delta, Omicron BA.1 and Omicron BA.2 variants were used to construct plasmids and express the proteins in glycoengineered *Pichia pastoris*. A stable 4 L-scale yeast fermentation and purification process was established to obtain high-purity RBD proteins with a complex glycoform N-glycosyl structure that was fucose-free. The RBD glycoproteins were combined with two adjuvants, Al(OH)_3_ and CpG, which mitigated the typical disadvantage of low immunogenicity associated with recombinant subunit vaccines. To improve the broad-spectrum antiviral activity of the candidate vaccine, Delta RBD proteins were mixed with BA.2 RBD proteins at a ratio of 1:1 and then combined with two adjuvants—Al(OH)_3_ and CpG—to prepare a bivalent vaccine. The bivalent vaccine effectively induced mice to produce pseudovirus-neutralizing antibodies against SARS-CoV-2 variants, Delta, Beta, and Omicron sublineages BA.1, BA.2, BA.5. The bivalent vaccine could neutralize the authentic wild-type SARS-CoV-2 strain, Delta, BA.1.1, BA.2.2, BA2.3, and BA.2.12.1 viruses, providing a new approach for improving population immunity and delivering broad-spectrum protection under the current epidemic conditions.

## 1. Introduction

The cumulative global impact of SARS-CoV-2 infections has caused over 600 million cases (https://COVID19.who.int/, accessed on 15 March 2023), posing a severe threat to public health [1]. The surface of SARS-CoV-2 features a trimeric spike (S) protein, comprising an S1 subunit bound to the host cell [2]. Upon binding of the receptor-binding domain (RBD) on the S1 subunit with the human cell surface receptor, angiotensin-converting enzyme 2 (hACE2), the S protein undergoes structural rearrangement. The S2 facilitates membrane fusion, enabling the virus to enter cell and subsequently initiate a sequence of physiological actions [3]. Because of the single-stranded RNA, SARS-CoV-2 exhibits obvious errors while replicating, subsequent to infiltrating human cells [4]. The diverse hosts of coronaviruses and their own genome structures make these viruses prone to gene mutation and genetic recombination [5]. This susceptibility is manifested as a rich genetic diversity, allowing some variants to escape pre-existing immunity and even become impossible to immunize against with the currently available SARS-CoV-2 vaccines [6,7]. According to the CDC available to the public (https://www.cdc.gov/coronavirus/2019-ncov/variants/variant-classifications.html, accessed on 15 March 2023), SARS-CoV-2 variants are classified as either variants being monitored (VBM) or variant of concern (VOC). The SARS-CoV-2 variants from Alpha to Kappa have large numbers of mutation sites, and are all VBMs [8]. Numerous Omicron subvariants, e.g., sublineages BA.1.1, BA.2.1, BA.5, have also emerged as VOCs [9]. Among them, BA.5 showed comparatively stronger transmission ability [6]. The existing SARS-CoV-2 vaccines antibodies show various degrees of decline in the effectiveness against Omicron and its subvariants [10]. Therefore, the development of multivalent vaccines is worth considering. As the primary neutralizing epitope, the RBD of the relevant S protein (RBD SARS-CoV-2) is an excellent candidate for designing vaccines [11,12].

In previous work, a glycoengineered *Pichia pastoris*, in which the mammalian capacity for *N*-glycosylation modifications is activated, was prepared by modifying the glycosylation pathway [13]. Glycoengineered, yeast-expressed vaccines, the SARS-CoV-2 RBD and H7N9 virus hemagglutination(HA) glycoprotein vaccines, have demonstrated favorable immunogenicity and protective efficacy [14]. A vaccine targeting the Omicron variants of it has also been developed. The serum obtained from mice immunized with an BA.2-specific vaccine demonstrated significantly lower neutralization efficacy against the Delta and Beta variants, compared to efficacy against the BA.2 variants. Expression of the RBD glycoproteins of the SARS-CoV-2 variants Delta, BA.1, and BA.2 in glycoengineered yeast, aided by Al(OH)_3_, resulted in virus-neutralizing antibodies generated for each variant in mice. We synthesized a bivalent vaccine based on SARS-CoV-2 Delta RBD vaccine and immunized mice with this bivalent vaccine. The results indicate that this prepared vaccine effectively induced cross-neutralizing immunity against various SARS-CoV-2 variants, including Alpha to Delta, and the Omicron sublineages. The bivalent vaccine demonstrated a superior broad-spectrum neutralizing activity compared with the monovalent vaccine, making it an effective strategy for achieving overall mucosal immunity against SARS-CoV-2 VBMs.

## 2. Materials and Methods

### 2.1. Bacterial Strains, Yeast Strains, Plasmids, and Other Materials

*E. coli* DH5α (TakaRa Biotech, Beijing, China) was cultivated in LB medium at 37 °C. Tryptone and yeast extract were obtained from OXOID (Thermo Scientific, Waltham, MA, USA); glucose was bought from Shanghai Chemical Reagent Co., Ltd. (Shanghai, China). The approach used for constructing glycoengineered *P. pastoris* was previously reported [14]. The glycoengineered *P. pastoris* strains were grown in YPD medium. Mice were purchased from Weitonglihua Experimental Animal Factory (Beijing, China). And the research contents were approved by the Institutional Animal Committee. The relevant welfare ethics number for this study is IACUC-2022-042.

### 2.2. RBD Gene Cloning and Protein Level of SARS-CoV-2 Variants

The RBD gene sequences of SARS-CoV-2 variants were obtained from the NCBI database, with the GenBank accession numbers, OM858819.1, OX315743.1, and OX315675.1, respectively. The SARS-CoV-2 Delta, BA.1, BA.2 RBD genes were prepared and then integrated into the pPICZαA vector at the XhoI/NotI sites, resulting in the creation of the expression plasmids pPICZαA-Delta, pPICZαA-BA.1 and pPICZαA-BA.2 RBD. The AOX I promoter regulates these plasmids, and the proper insertion was verified through PCR and sequencing analysis using 5′-AOX forward primers (5′-GACTGGTTCC AATTGACAAGC-3′) and 3′-AOX (5′-GGCAAATGGCATTCTGACAT-3′) primers. *Bgl*II-linearized pPICZαA-Delta, pPICZαA-BA.1, and pPICZαA-BA.2 RBD were each transformed int *P. pastoris*, and the resulting yeasts were named Glycoeng-yeast/Delta RBD, Glycoeng-yeast/BA.1 RBD, and Glycoeng-yeast/BA.2 RBD.

### 2.3. Strain Fermentation and the Purification Procedure for the RBDs

The fermentation process was performed in a 4-l bioreactor in three phases: the basal cultivation phase, the feeding culture phase, and the induction stage. To initiate the basal culture phase, 200 mL of RBD seed solution was quickly poured along with individually sterilized ingredients into the fermenter inoculation port. The addition of NH_3_•H_2_O was terminated 36 h before the start of the basal incubation phase after the level of dissolved oxygen had increased. To enhance yeast density, the yeast was fed in batches with 400 mL of 5% yeast extract and 10% tryptone. Oxygen levels increased after 12 h of incubation. During the induction of the culture phase, 500 mL of 2 M sorbitol, 500 mL of 10% yeast extract, and 150 mL of methanol were added induce RBD expression. The cultivation process was terminated after 48 h of further fermentation.

The Glycoeng-yeast/Delta RBD, Glycoeng-yeast/BA.1 RBD, and Glycoeng-yeast/BA.2 RBD cells were collected via centrifugation for half an hour after being cultured and fermented. The supernatant containing the secreted RBD proteins was used for purification. The RBD samples underwent a series of purification steps, including multimodal cation exchange using a SP-Big Bead column (GE Healthcare, Pittsburgh, PA, USA), hydrophobic chromatography, strong anion exchange using a Source 30Q column, and finally, size exclusion chromatography. Highly pure RBD protein was finally obtained using a blocking chromatography column (Superdex-75) (GE Healthcare, Pittsburgh, PA, USA).

### 2.4. Feature of Recombinant SARS-CoV-2 RBD Proteins

Recombinant protein expression was evaluated via SDS-PAGE with 12% polyacrylamide gel and subsequent staining with GelCode Blue dye. The SEC-HPLC column TSKgel G2000 SWXL (Merck, Darmstadt, Germany) was calibrated with molecular weight standards (GE Healthcare) using an Agilent Technologies 1260 Infinity HPLC (Agilent Technologies Inc., Palo Alto, CA, USA). Absorbance values were recorded at 280 nm.

### 2.5. Vaccination of Mice and Identification of Antibodies against Recombinant RBD in Mice Serum

Female BALB/c mice aged 6–8 weeks were assigned into eight groups (*n* = 10). Blood samples were collected from pre-immunized mice as blank controls in the ELISA test for antibody titer determination, and we set up three repeat holes. The recombinant RBD protein vaccines were each administered intramuscularly with two adjuvants—100 µg of Al(OH)_3_ (CRODA, Elsenbakken, Denmark) and 50 µg of CpG2006 (TGCTCGTTTTGTGCTTTTGTGCTT)—for immunization. In our previous study, two immunizations of RBD-WT at days 0 and 14; at days 0 and 21; at days 0 and 28; and three immunizations at days 0, 14, and 28 were studies. The results indicate that the “0, 14 d,” “0, 21 d,” or “0, 14, 28 d” schedules could be potential immunization strategies [13]. Mice were immunized with 100 μL (intramuscular injection) in their hind leg muscles on days 0 and 14. The first batch of animal experiments were 10 µg of WT RBD (group 1) and 10 µg of Delta RBD (group 2). Then, the second batch of vaccine immunogens in groups 1–7 were as follows: 10 µg of WT RBD (group 1); 10 µg of BA.1 RBD (group 2); 10 µg of BA.2 RBD (group 3); 5 µg of BA.1 RBD/5 µg of BA.2 RBD (group 4); 10 µg of Delta RBD (group 5); 10 µg of Delta RBD/10 µg of BA.2 RBD (group 6); 5 µg of Delta RBD/5 µg of BA.2 RBD (group 7). Group 8 was immunized with only the adjuvants, Al(OH)_3_/CpG.

Blood samples were collected from experiment animals on day 28 after vaccination. Following 1–2 h of coagulation at 25 °C, the blood samples were centrifuged at 3000 rpm for 10 min at 4 °C. The supernatant obtained was collected. Recombinant RBD was applied to 96-well plates and left overnight at 4 °C. Subsequently, the plates underwent three rounds of washing with 0.1% Tween-20 in PBS (PBST), followed by the addition of PBST blocking solution, which contained 5% skim milk. Incubation at 25 °C was performed for 1 h. After adding serial dilutions, incubated at 37 °C for 1 h. Subsequently, the plates underwent three rounds of washing with PBST. The addition of antibodies to the wells involved diluting goat anti-mouse IgG HRP-conjugated antibody and other antibodies at a ratio of 1:5000. The amount added to each well was 100 µL. The plates were incubated at 25 °C for 1 h, followed by five rounds of washing with PBST. The next step involved incubating the plates with 3,3′,5,5′-tetramethylbiphenyl diamine for 10 min. The reaction was terminated with 2.0 M H_2_SO_4_ termination solution. Absorbance was detected at 450 nm. To measure the titers of the recombinant protein-induced RBD antibodies, serially diluted serum samples were analyzed via titration.

### 2.6. Pseudovirus Neutralization Assay

Antibody-mediated inhibition of the pseudo-neutralization of 293T-ACE2 cells was conducted using the serum samples with SARS-CoV-2 pseudoviruses. The samples were heated at 56 °C for 0.5 h. Then, each of the following pseudoviruses was incubated with serially diluted samples for 1 h: SARS-CoV-2-Fluc Beta; Delta, Omicron BA.1; Omicron BA.2; Omicron BA.2.12.1; and Omicron BA.4/5. Finally, the virus–antibody mixture was transferred to 96-well plates that had been pre-coated. After 8 h, we added fresh medium, and after 2 days, 100 μL luciferase assay substrate (Vazyme) was added, following the removal of an equivalent volume of medium. After shaking for 2 min, the plates were incubated for 3 min, and the fluorescence signal was detected based on a multimode plate reader. The ED_50_ values were determined by performing nonlinear regression calculations with the aid of GraphPad Prism 8 [15].

### 2.7. Authentic Virus-Neutralizing Assay

The authentic SARS-CoV-2 experiments were conducted at the Xiamen Center for Disease Control. To assess the neutralizing effects of the serum antibodies induced by the subunit vaccines generated from various SARS-CoV-2 variant sequences, we used SARS-CoV-2 WT, Delta variant, and four Omicron variants. Briefly, 96-well cell culture plates were inoculated with 2 × 10^4^ Vero-E6 cells in 200 μL of DMEM medium containing 10% FBS and incubated at standard environment overnight. After initiating the incubation at 37 °C for 1 h, serum samples (starting dilution of 1:50) were serially diluted three times and combined with 500 TCID_50_ of SARS-CoV-2. After the incubation, the cell culture medium in the 96-well plate was discarded, and the prepared serum-virus mixture was added into the experimental wells for adsorption at standard condition for 1 h. The plates were rinsed twice with sterile PBS, and then DMEM medium containing 2% FBS was added. The cells were incubated under standard conditions for 5 days. On day 4, the cytopathic effect (CPE) was observed using a microscope, and the number of wells with CPE was recorded. Two replicates of each concentration of diluted serum were tested. The Karber method was used to determine the neutralizing titer of each serum sample. Blank controls with no serum-virus sample, and virus controls containing only viruses, were included in the experiment.

## 3. Results

### 3.1. Expression of the RBD Proteins of SARS-CoV-2 Variants in Glycoengineered P. pastoris

**Figure 1:**
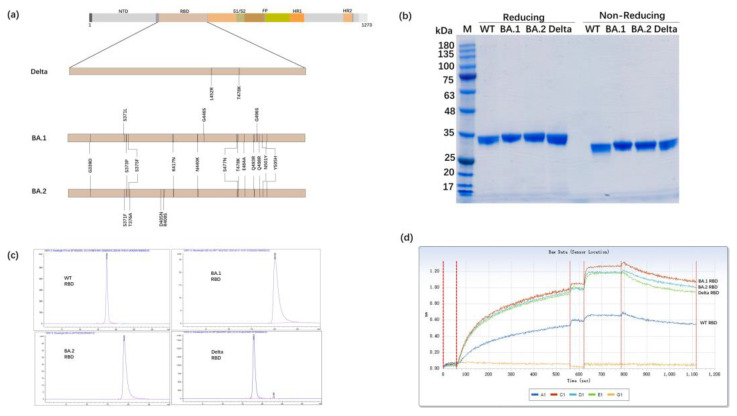
Analysis of the RBD domain in the spike protein of variants. (**a**) The location of the RBD. The mutations in the SARS-CoV-2 Delta, Omicron BA.1, BA.2 variants are marked. The Delta RBD contains two mutation sites (L452R and T478K), the Omicron BA.1 RBD contains 15 mutation sites, and the Omicron BA.2 RBD contains 16 mutation sites, relative to SARS-CoV-2 WT RBD. (**b**) The SDS-PAGE results of the RBD protein from SARS-CoV-2 WT, BA.1, BA.2, and Delta. WT: SARS-CoV-2 wildtype RBD; BA.1: variant BA.1 RBD; BA.2: variant BA.2 RBD; and Delta: variant Delta RBD. The purified target protein showed a uniform band via both reduced and non-reduced SDS-PAGE. (**c**) The SEC-HPLC results of purified SARS-CoV-2 WT, BA.1 RBD. (**d**) The affinity of the SARS-CoV-2 WT, Delta, BA.1, RBD to ACE2.

Figure 1a illustrates the position of the RBD on the SARS-CoV-2 S protein. The RBD gene fragment specific to each SARS-CoV-2 variant was incorporated into the pPICZαA vector by placing it within NotI sites. The glycoengineered *P. pastoris* was used to secrete the RBD protein into the supernatant, which was subsequently purified using multiple chromatography techniques, including cation exchange, hydrophobic, anion and cation chromatography, and gel filtration. The purified target protein showed a consistent band in reduced and non-reduced SDS-PAGE (Figure 1b). The SEC-HPLC method was used to assess the purity of the RBD protein of SARS-CoV-2 WT, BA.1, and Delta variants, and the results indicate that the purity of each was >98% (Figure 1c). The binding abilities between ACE2 and each of the RBD proteins from the different SARS-CoV-2 variants were found to be similar, and the order from strongest to weakest binding was as follows: BA.2 RBD > BA.1 RBD > Delta RBD > WT RBD (Figure 1d, Table S1). This result may be related to the relatively higher transmission of the variants with stronger binding affinity.

### 3.2. Antibody Titers after Two Immunizations with the Bivalent RBD Vaccine

**Figure 2:**
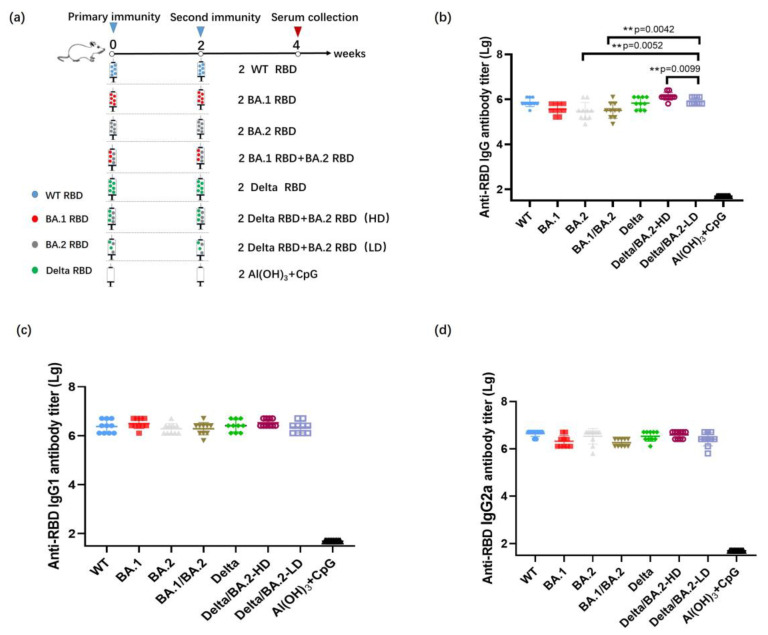
Humoral immune responses elicited by immunization with RBD vaccines in BALB/c mice. (**a**) The timeline for analyzing the humoral immune responses of BALB/c mice to an RBD vaccine. Mice received two intramuscular injections on days 0 and 14. The serum was collected on day 28 after the initial immunization. (**b**) Titers of antibodies after vaccination with vaccines containing the RBDs of the specified SARS-CoV-2 variants. (**c**,**d**) Specific IgG1 (**c**) and IgG2a (**d**) antibody titers after immunization with a vaccine containing RBD from the indicated SARS-CoV-2 variant. Statistical significance was analyzed based on *t*-tests; ** *p* < 0.01.

Previous research demonstrated that the antibody titers induced by SARS-CoV-2 RBD vaccines reached their peak value at 4 weeks after vaccination. Therefore, mouse serum was collected for the detection of RBD-binding antibodies on day 28 after the initial immunization (Figure 2a). Immunization with 10 µg of the RBD monovalent vaccine and 10 and 20 µg of the RBD bivalent vaccine induced SARS-CoV-2-specific IgG antibody titers greater than 3.02 × 10^5^, 3.24 × 10^5^, and 1.38 × 10^6^, respectively. (Figure 2b). Antibody typing of specific antibodies in mouse serum showed that the RBD bivalent vaccine could induce robust both IgG1 and IgG2a antibodies (Figure 2c,d). In our previous study, we found that antibody typing of mice sera showed that the RBD-WT and RBD-Beta bivalent vaccine elicited robust IgG1, IgG2a, IgG2b, and IgG3 responses, and spleen cells from mice 10 days after the second immunization were removed and assayed for IFN-γ and IL-2 secreted by Th1 cells and for IL-4 secreted by Th2 cells using ELISpot. IFN-γ and IL-2 were higher, and IL-4 was lower in the adjuvant CpG group than in the non-CpG group, suggesting that the vaccine may have induced cytokine levels more in favor of the Th1 response [16].

### 3.3. Pseudovirus-Neutralizing Antibody Titers

**Figure 3:**
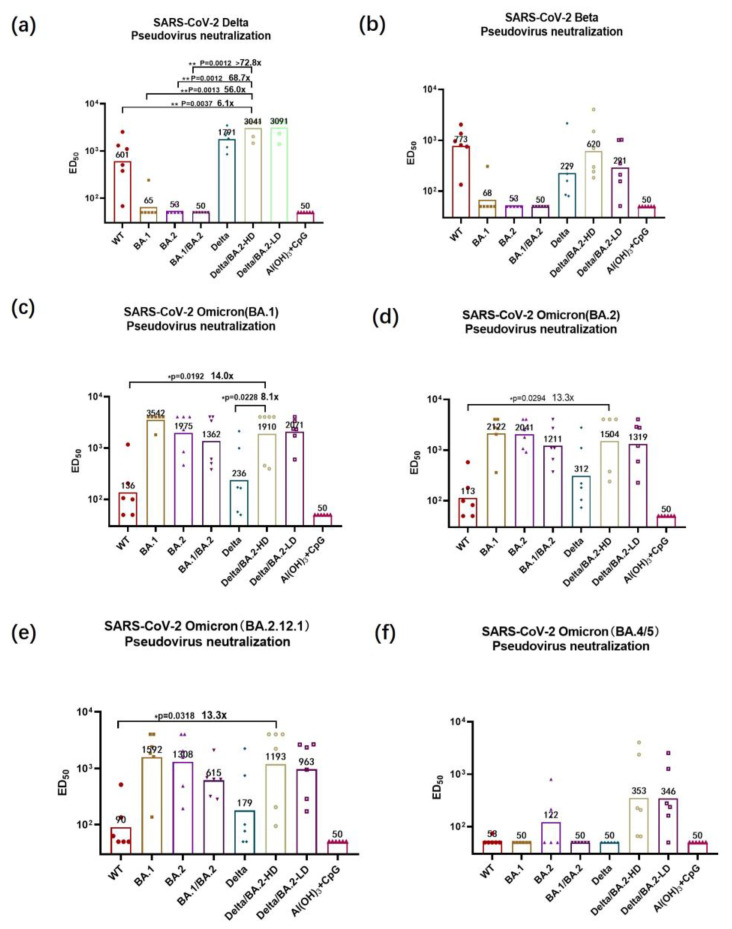
The neutralizing activity against SARS-CoV-2 pseudoviruses (variants: Delta; Beta; BA.1; BA.2; BA2.12.1; and BA.4/5) was assessed in six mice vaccinated with recombinant S-RBD proteins from different SARS-CoV-2 variants. (**a**–**f**) Neutralizing activity of serum from S-RBD-immunized mice against pseudoviruses of SARS-CoV-2 Delta (**a**), Beta (**b**), BA.1 (**c**), BA.2 (**d**), BA2.12.1 (**e**), and BA.4/5 (**f**). Statistical significance was analyzed via *t*-tests; * *p* < 0.05, ** *p* < 0.01.

The vaccine-induced neutralizing antibody titer is an important indicator of vaccine effectiveness. The titers of neutralizing antibodies against wild-type (WT) SARS-CoV-2, BA.1 pseudoviruses of the RBD-Delta group was higher than the RBD-WT group (Figure S1). To assess the neutralizing effects of the antibodies produced in the immunized group, we employed various SARS-CoV-2 pseudoviruses, including Beta and Omicron (specifically, BA.1, BA.2, BA2.12.1). The neutralizing antibody titers induced by the BA.1/BA.2 bivalent vaccines for the Delta pseudovirus did not differ significantly from that of negative group, indicating that the Omicron RBD was unable to generate a neutralizing antibody that provided cross-protection against the Delta variant. For the neutralizing antibodies against the Delta pseudovirus, the median effective dilution 50% (ED_50_) value of the group given the high-dose Delta/BA.2 RBD bivalent vaccine was 1:3041 (Figure 3a). The neutralizing antibody titer induced in the high-dose Delta/BA.2 RBD group was obviously higher compared the SARS-CoV-2 WT, BA.1 or BA.1/BA.2 groups (*p* < 0.01). The BA.1 and BA.1/BA.2 bivalent vaccines were virtually ineffective in generating a neutralizing antibody that could against the SARS-CoV-2 Beta variant pseudovirus. But, the ED_50_ value for the high-dose Delta/BA.2 RBD bivalent vaccine group for the neutralizing antibody against the Beta pseudovirus was 1:620 (Figure 3b). Compared with the WT group, the high-dose Delta/BA.2 bivalent group exhibited a 14.0-fold increase in neutralizing antibody effectiveness against the BA.1 pseudovirus, and was 8.1-fold higher than the Delta RBD vaccine group (Figure 3c). For the neutralizing antibody against SARS-CoV-2 BA.2 pseudovirus, the ED_50_ value for the high-dose Delta/BA.2 bivalent vaccine group was 8.1-fold higher than for the WT RBD vaccine group (Figure 3d). The ED_50_ value for the high-dose Delta/BA.2 bivalent vaccine group for the neutralizing antibody against the BA.2.12.1 pseudovirus was 1:1193 (which was 13.3-fold higher than that of the WT RBD vaccine group) (Figure 3e). The high-dose Delta/BA.2 bivalent immune group had an ED_50_ value for that against the BA.4/5 pseudovirus of 1:353 and could induce an effective neutralizing antibody. There were no significant differences between the low-dose and high-dose vaccination groups in either IgG antibody titers or neutralizing activity. Therefore, the Delta/BA.2 bivalent vaccine demonstrated a greater range of neutralizing activity against multiple VBMs, compared with the other tested vaccines.

### 3.4. Authentic Virus-Neutralizing Antibody Titer Determination

**Figure 4:**
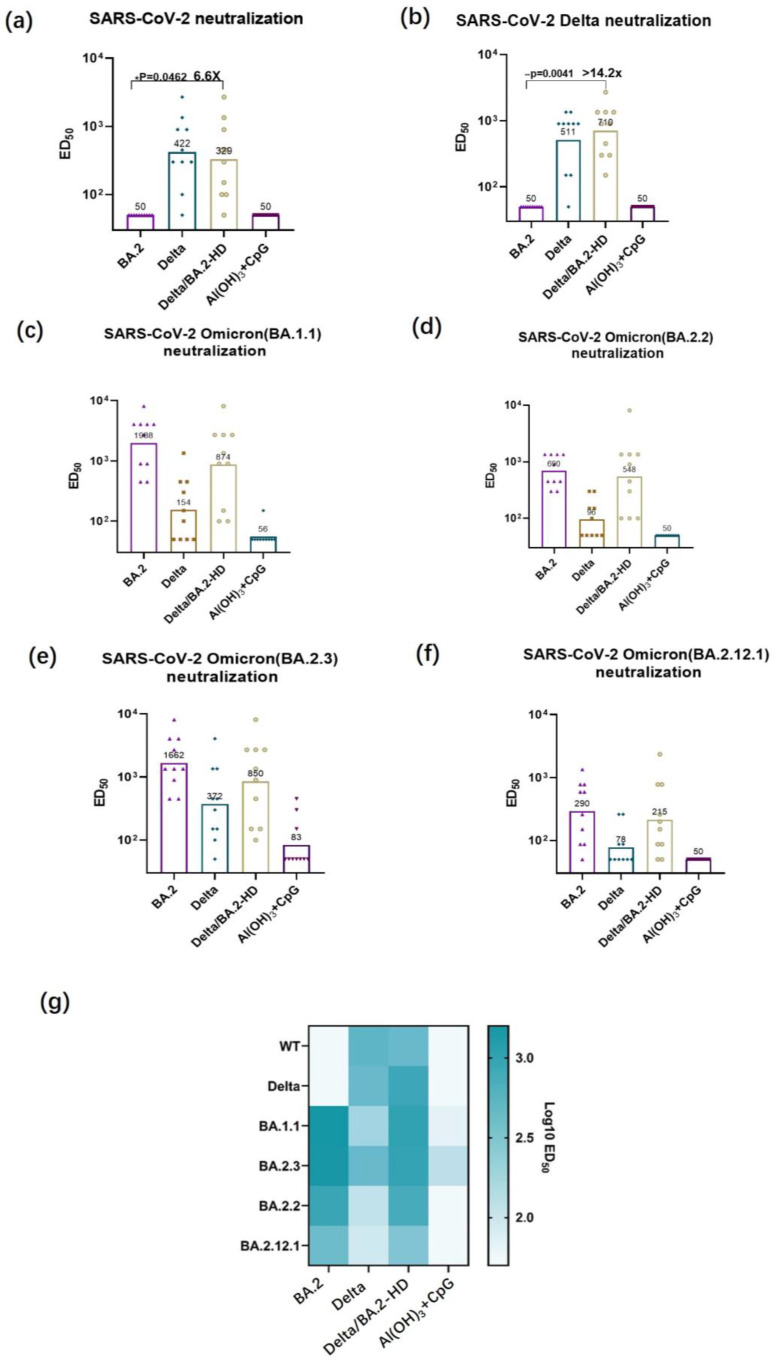
Neutralizing activity against authentic viruses (SARS-CoV-2 WT, Delta, BA.1.1, BA.2.2, BA2.3, BA2.12.1). (**a**–**f**) Neutralizing activity against the authentic viruses, SARS-CoV-2 WT (**a**), Delta (**b**), BA.1.1 (**c**), BA.2.2 (**d**), BA2.3 (**e**), and BA.2.12.1 (**f**). (**g**) Heatmap of authentic virus neutralizing antibody titers. Statistical significance was determined using *t*-tests; * *p* < 0.05, ** *p* < 0.01.

Following the immunization of mice with a high dosage of the Delta/BA.2 RBD bivalent vaccine, the variants reached elevated levels. The Delta/BA.2 bivalent vaccine group displayed a significantly higher ED_50_ value of 1:329 for that against SARS-CoV-2 WT, compared with the BA.2 vaccine group, which had an ED_50_ value of 1:50 (Figure 4a). In addition, the Delta/BA.2 bivalent vaccine group displayed a 14.2-fold increase for the neutralizing antibody against the SARS-CoV-2 Delta variant, compared to BA.2 vaccine group (ED_50_ = 1:710 and 1:50, respectively) (Figure 4b). The ED_50_ value for the neutralizing antibody against the BA.1.1 subvariant reached 1:874, and the titers for this virus-neutralizing antibody also reached a high level (Figure 4c). The ED_50_ values for the neutralizing antibodies against the subvariants, BA.2.2 and BA.2.3, and BA.2.12.1, reached 1:548, 1:850, and 1:215, respectively (Figure 4d–f). Thus, immunization using the Delta/BA.2 vaccine resulted in broad neutralization of WT, Delta variant, relevant Omicron subvariants BA.1.1, and BA.2.12.1, which was superior to immunization with the monovalent RBD vaccines (Figure 4e).

## 4. Discussion

The glycoengineered yeast production platform allows for a rapid response to virus mutations and scalable production [13,17]. Thus, a candidate vaccine can be prepared in a process that is expected to provide stable production in a few weeks. The L452R mutation in the RBD of S protein can interfere with the binding reaction between the anti-S protein antibody and the RBD; thus, strengthening the ability for immune escape. Additionally, the T478K mutation in the RBD of the Delta variant S protein strengthens the affinity between the RBD and ACE2, facilitating virus invasion into the host cell [18]. A previous study has shown that the SARS-CoV-2 Delta RBD vaccine elicited broadly neutralizing antibodies [19]. The existing vaccines and the antibodies that they induce show various degrees of decline in effectiveness against Omicron and its subvariants [20]; our developed Delta and Omicron BA.2 bivalent vaccine can provide better broad-spectrum protection. In the present study, the serum from mice immunized with Delta and Omicron BA.2 RBD not only contained higher titers of neutralizing antibodies but also had a broad ability for neutralizing variants of pseudovirus, compared with the serum from mice immunized with the RBD of a single variant. Thus, our approach provides a new strategy for epidemic prevention and control. However, the vaccine compatibility method that we adopted here was 1:1. To address the continuous emergence of novel coronavirus mutants, we will carry out in-depth research on the ideal proportions of S-RBD from different SARS-CoV-2 variants for an excellent vaccine. The SARS-CoV-2 Delta and Omicron BA.2 vaccine produced from glycoengineered yeast described here is expected to be a candidate for a broad-spectrum vaccine against SARS-CoV-2.

BALB/c mice typically respond to subunit vaccines with a Th2-type immune response, which is associated with the stimulation of IgG1 antibodies, inducing cellular immune response. However, the major antibody isotype present in the sera of mice that survive viral infections is IgG2a, which is stimulated during Th1-type immune responses, inducing humoral immune response [21]. Antibody typing of specific antibodies in mouse serum showed that the RBD bivalent vaccine could induce robust both IgG1 and IgG2a antibodies. The specific IgG1 and IgG2a antibody titers induced by Delta-Omicron bivalent subunit vaccine were over 10^6^. This indicates that both humoral and cellular immunity are induced.

Antibody responses against the SARS-CoV-2 pseudovirus and authentic variants were compared. The Delta pseudovirus neutralizing antibody titer induced in the high-dose Delta/BA.2 RBD group was 68.7-fold higher than the BA.2 group (*p* < 0.01), and the corresponding groups antibody titer of authentic Delta virus was 14.2-fold higher. The neutralizing antibody titers of the authentic viruses were generally lower than the pseudoviruses variants; therefore, the differences of neutralizing antibodies against authentic viruses were smaller than that of the pseudovirus. The BA.1 pseudovirus neutralizing antibody titer induced in the high-dose Delta/BA.2 RBD group was 8.1-fold higher compared the Delta RBD group (*p* < 0.01), but the antibody titers of the Delta/BA.2 RBD group and the Delta RBD group against authentic BA.1 virus had no significant differences. The BA.2 and BA2.12.2 pseudovirus neutralizing antibody titers had no significant differences between Delta/BA.2 RBD group and Delta RBD, which is consistent with authentic viruses.

In this study, we used glycoengineered Pichia pastoris to express SARS-CoV-2 variants RBD. In previous study, the H7N9 influenza virus, hemagglutinin (HA), subunit particle vaccine prepared by means of glycosylation-modified humanized yeast, which has mammalian *N*-glycosylation ability, has been shown to have good immunogenicity and protected mice from the H7N9 virus. The limitation of glycoengineered yeast production for mass vaccines may be a low expression of proteins with complex structures [14].

The emergence of new SARS-CoV-2 variants driven by immune pressure seriously threatens human health. Updating current vaccines can increase the breadth of neutralization and protect against the new variants, such as boosting with Moderna’s bivalent mRNA-1273.214 or mRNA-1273.222 vaccines, enhanced protection against BA.1 and BA.4/5, respectively [22]. This study demonstrated that RBD-based bivalent subunit vaccines can also provide potent protection against corresponding variants, although full-length, spike-based vaccine is preferable, and glycoengineered yeast provides a new platform to upgrade current RBD-based subunit vaccines, which can be produced in one month and which possess an excellent safety profile.

## 5. Conclusions

Immunization using the Delta/BA.2 vaccine combined with two adjuvants—Al(OH)_3_ and CpG—effectively induced mice to produce pseudovirus-neutralizing antibodies against SARS-CoV-2 variants, Delta, Beta, and Omicron sublineages BA.1, BA.2, BA.5. The bivalent vaccine could neutralize the authentic wild-type SARS-CoV-2 strain, Delta, BA.1.1, BA.2.2, BA2.3, and BA.2.12.1 viruses, providing a new approach for improving population immunity and delivering broad-spectrum protection under the current epidemic conditions. The bivalent vaccine effectively induced cross-neutralizing immunity against various SARS-CoV-2 variants, including Alpha to Delta, and the Omicron sublineages, making it an effective strategy for achieving overall mucosal immunity against SARS-CoV-2 VBMs.

## Data Availability

The raw data supporting the conclusions of this article will be made available by the authors, without undue reservation.

## Supplementary Materials

The following supporting information can be downloaded at https://www.mdpi.com/article/10.3390/vaccines11101539/s1, Figure S1: Neutralizing activity against the pseudoviruses (SARS-CoV-2 WT, Beta, Delta, BA.1) following vaccination with WT-RBD and Delta-RBD. *p*-values were analyzed with *t* tests; *: *p* < 0.05, **: *p* < 0.01, ****: *p* < 0.0001; Table S1: Affinity constant of the WT, Delta, BA.1, or BA.2 RBD to ACE2.
