# Supplementary material for: A Delta–Omicron Bivalent Subunit Vaccine Elicited Antibody Responses in Mice against Both Ancestral and Variant Strains of SARS-CoV-2
Source: Vaccines (Basel). 2023 Sep 28;11(10):1539. doi: 10.3390/vaccines11101539 (PMC10611268; doi:10.3390/vaccines11101539)
Supplement: Supplementary file 1 [file vaccines-11-01539-s001.zip › vaccines-2554834-supplementary.pdf]

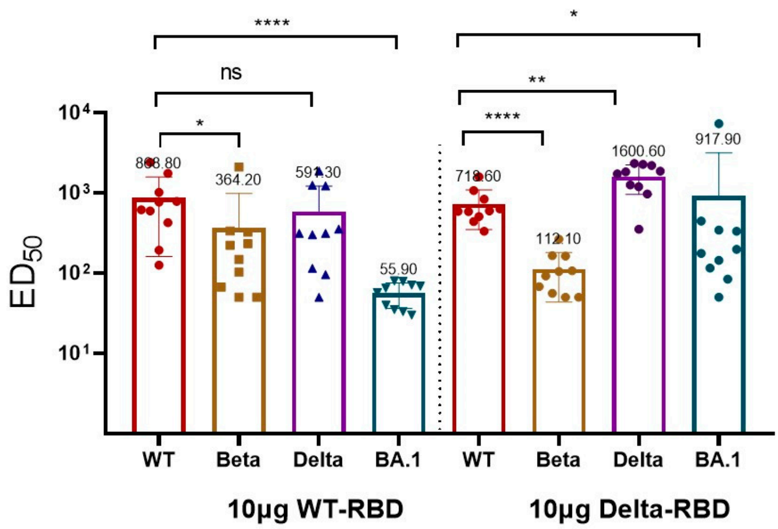

Figure S1. Neutralizing activity against the pseudoviruses (SARS-CoV-2 WT, Beta, Delta, BA.1) following vaccination with WT-RBD and Delta-RBD. *p*-values were analyzed with *t* tests; *: $p < 0.05$, **: $p < 0.01$, ****: $p < 0.0001$.

Table S1. Affinity constant of the WT, Delta, BA.1, or BA.2 RBD to ACE2.

Assoc. (Sample) Loc.	Sample	K_D(10^{-8} M)
A3	WT	43.8
C3	BA.1	3.1
D3	BA.2	2.4
E3	Delta	16.1
